# Supplementary figures and images for: Dynamics of pax7 expression during development, muscle regeneration, and in vitro differentiation of satellite cells in rainbow trout (Oncorhynchus mykiss)
Source: PLoS One. 2024 May 8;19(5):e0300850. doi: 10.1371/journal.pone.0300850 (PMC11078358; doi:10.1371/journal.pone.0300850)

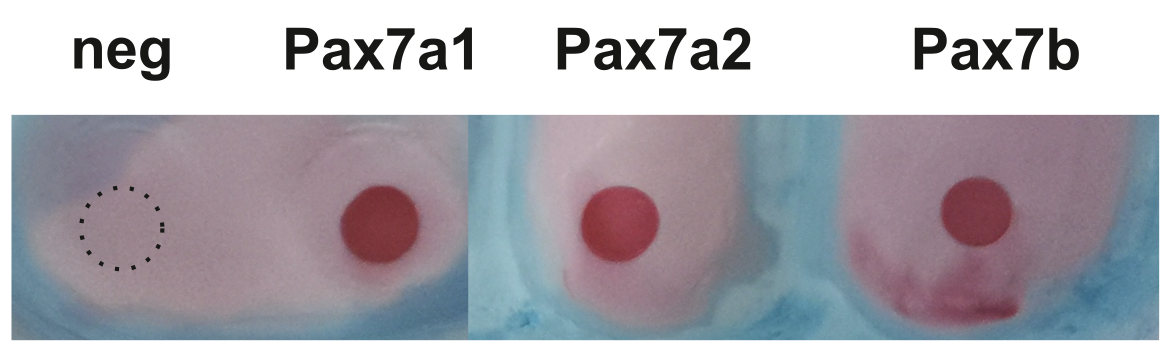

Supplement: S1 Fig — We developed a dot-blot hybridization approach in order to determine if the pax7 probe set is able to target the three pax7 genes present in the trout genome (pax7a1, pax7a2, pax7b) (Bio-Techne, Om-pax7b-cust # 575461). First, we transcribed cRNA sens of pax7a1, pax7a2 and pax7b from pax7 synthesized genes (Eurofins Genomics), using appropriate polymerase (Proméga, Riboprobe Systems sp6, # P1420, Riboprobe Systems T7, # P1440). The length of the cRNA is about 900 nt. We then applied 1μl (50ng) of each pax7 cRNA onto a nitrocellulose membrane (Macherey-Nagel) and performed a dot-blot hybridization analysis. After UV fixation (UV crosslinker, Appligene Oncor), blots were incubated for 1 hour at 40°C with 1 drop of pax7 probe set in order to hybridize with cRNA targets. After washing away excess probe, the presence of each pax7 cRNA was detected using a chromogenic RNAscope kit (Bio-Techne, RNAscope 2.5 HD Reagent Kit–RED, # 322350). Red dots indicate that the pax7 probe set recognizes the three pax7 and doesn’t recognize the negative control corresponding to the pax7a1 antisense RNA. (TIF) [file pone.0300850.s001.tif]

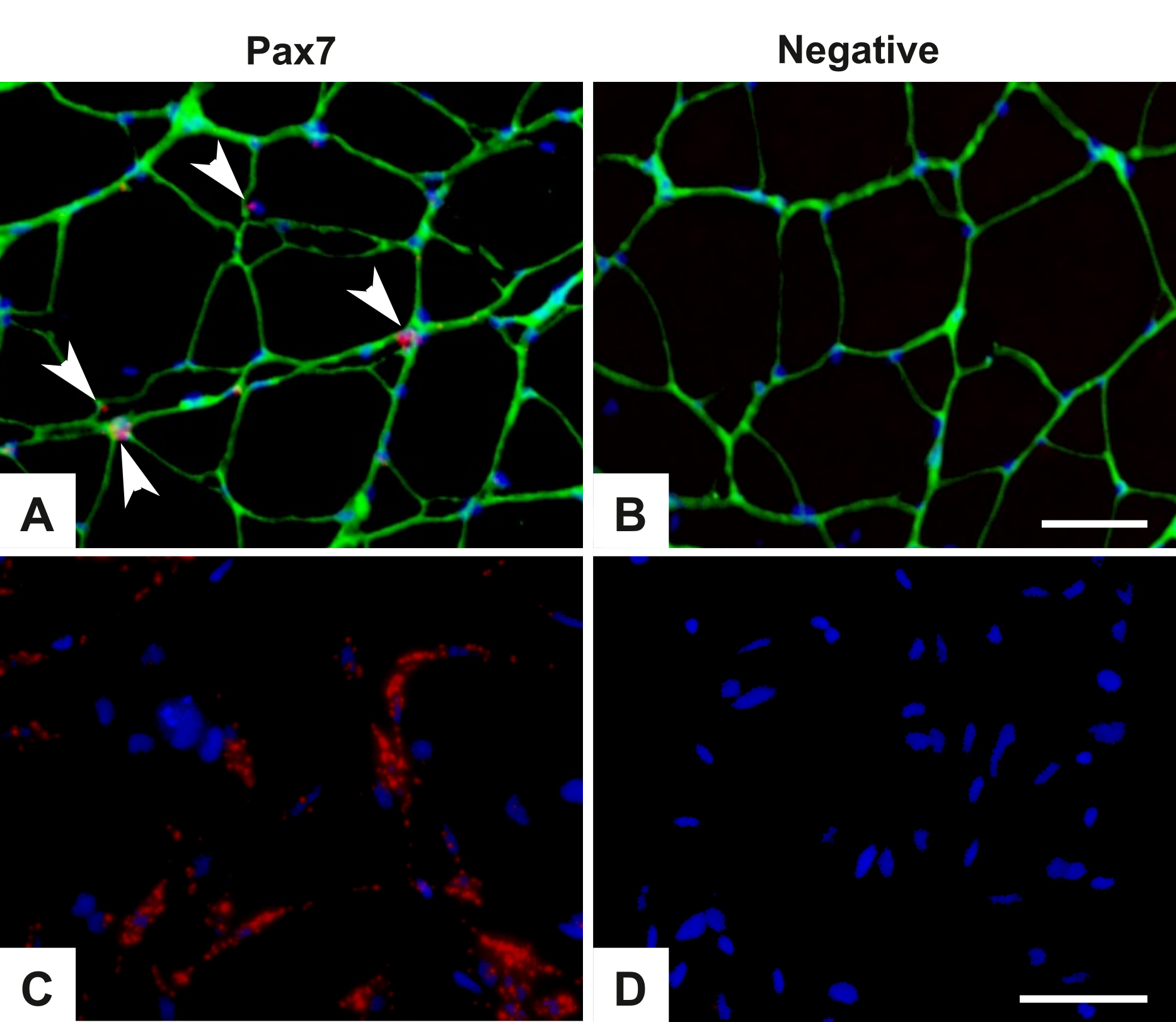

Supplement: S2 Fig — Transverse sections (A, B) of trout white muscle (10g) were analyzed by in situ hybridization for pax7 (red) and extracellular matrix was stained with Alexa 488-conjugated wheat germ agglutinin (green). Arrowheads indicate pax7+ cells beneath the basal lamina of the muscle fibers. No signal was observed with the negative probe against a bacterial gene DapB (B). Myogenic progenitors (C, D) cultured for 1 day in culture medium (DMEM, 10% SVF) were analysed by in situ hybridization for pax7 (red). A strong signal was observed in mononucleated cells (C). No signal was observed with the negative probe against a bacterial gene DapB (D). The nuclei are counterstained with DAPI and the scale bar corresponds to 50 μm. (TIF) [file pone.0300850.s002.tif]

Pax7a1 antisens  
cRNA (neg)

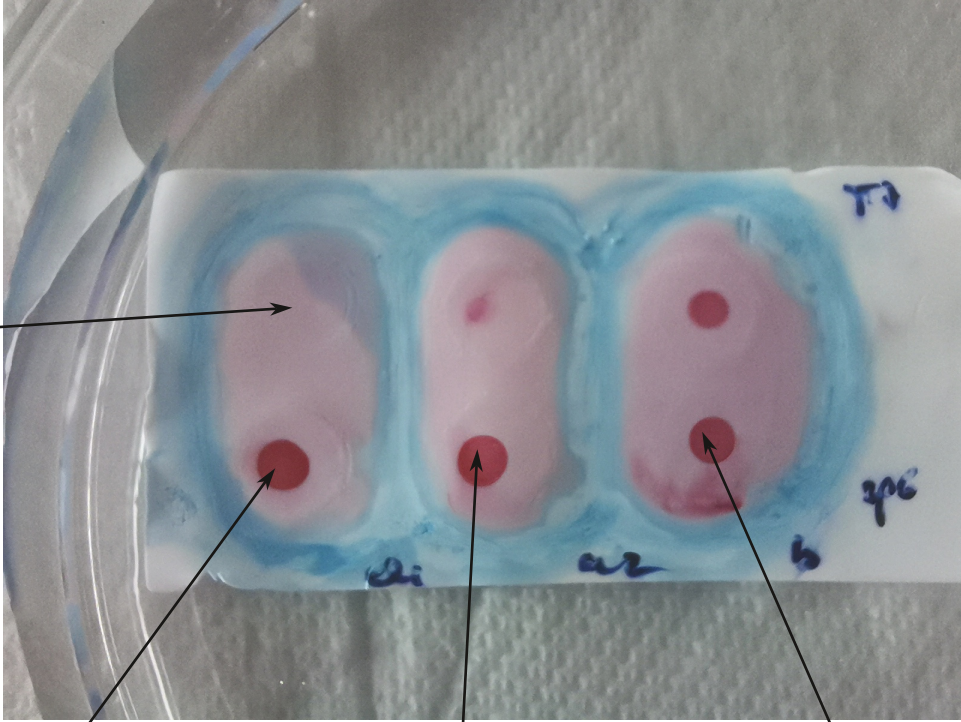

Pax7a1 sens cRNA

Pax7a2 sens cRNA

Pax7b sens cRNA

Supplement: S1 Raw images — We transcribed cRNA sens and antisens of pax7a1, pax7a2 and pax7b from pax7 synthesized genes (Eurofins Genomics), using appropriate polymerase (Proméga, Riboprobe Systems sp6, # P1420, Riboprobe Systems T7, # P1440). We then applied each pax7 cRNA onto a nitrocellulose membrane (Macherey-Nagel) and performed a dot-blot hybridization analysis. (PDF) [file pone.0300850.s003.pdf]
